# Stress and Its Effects on Glucose Metabolism and 11**β**-HSD Activities in Rats Fed on a Combination of High-Fat and High-Sucrose Diet with Glycyrrhizic Acid

**DOI:** 10.1155/2013/190395

**Published:** 2013-03-18

**Authors:** Hamish Alexander Fernando, Hsien-Fei Chin, So Ha Ton, Khalid Abdul Kadir

**Affiliations:** Monash University Sunway Campus, Jalan Lagoon Selatan, Bandar Sunway, 46150 Selangor Darul Ehsan, Malaysia

## Abstract

Chronic stress has been shown to have a strong link towards metabolic syndrome (MetS). Glycyrrhizic acid (GA) meanwhile has been shown to improve MetS symptoms caused by an unhealthy diet by inhibiting 11**β**-HSD 1. This experiment aimed to determine the effects of continuous, moderate-intensity stress on rats with and without GA intake on systolic blood pressure (SBP) across a 28-day period, as well as glucose metabolism, and 11**β**-HSD 1 and 2 activities at the end of the 28-day period. Adaptation to the stressor (as shown by SBP) resulted in no significant defects in glucose metabolism by the end of the experimental duration. However, a weakly significant increase in renal 11**β**-HSD 1 and a significant increase in subcutaneous adipose tissue 11**β**-HSD 1 activities were observed. GA intake did not elicit any significant benefit in glucose metabolism, indicating that the stress response may block its effects. However, GA-induced improvements in 11**β**-HSD activities in certain tissues were observed, although it is uncertain if these effects are manifested after adaptation due to the withdrawal of the stress response. Hence the ability of GA to improve stress-induced disturbances in the absence of adaptation needs to be investigated further.

## 1. Introduction

Metabolic syndrome (MetS) is an aberrance of metabolic functions resulting in abnormalities that are major risk factors for the development of cardiovascular disease (CVD) and type 2 diabetes mellitus (T2DM) [1]. Studies have indicated that there has been a significant increase in the frequency of MetS over the past 50 years, and this has been accounted to both the increased consumption of westernized diets and decreased physical activity [2]. Westernized diets are to a great extent high in fat and/or sugar, making these varieties of food highly obesogenic [2]. An experiment conducted by Panchal et al. [3] on male Wistar rats showed that a combined high-carbohydrate/high-fat diet (which accurately simulates the dietary intake of the average person) for 16 weeks caused an increase in body weight, energy intake, and abdominal fat deposition. This occurred due to impaired glucose tolerance, dyslipidaemia, and hyperleptinaemia and hyperinsulinaemia in the rats (hyperleptinaemia occurs as a result of leptin resistance in a similar way as hyperinsulinaemia occurs with insulin resistance). These were accompanied by damage to the heart, liver, and pancreas. 

Stress is an unavoidable aspect in today's world and hence plays an important part in our day to day lives. “Stress” can be defined as a disruption in the normal homeostatic functions of an organism caused by a “stressor”—a physiological or psychological challenge [4]. The “stress response” is a series of physiological and behavioural changes (referred to as “allostasis”) involving the HPA axis and the sympathetic nervous system that helps the organism to cope with these challenges [5, 6]. This is also referred to as the “fight or flight” response. 

However, chronic oversecretion of stress mediators, for example glucocorticoids (GCs) such as cortisol, as well as catecholamines, may result in problems such as hyperinsulinaemia and growth hormone and sex steroid hyposecretion, leading to visceral adiposity, loss of muscle (sarcopenia), arterial hypertension, glucose intolerance, and dyslipidemia, and therefore, MetS [7–10]. Visceral adiposity eventually leads to IR, while elevated cortisol and catecholamine concentrations, as well as low sex steroid levels, antagonize the effects of insulin and also increase blood glucose concentration independent of their effects on insulin. Put together, these effects play a major role in the development of MetS and diabetes mellitus [11].

The 11*β*-hydroxysteroid dehydrogenases (11*β*-HSDs) enzymes are involved in the regulation of the level of active GCs in the tissues [12]. There are two isoforms, 11*β*-HSD type 1 and 2. The two isoforms of 11*β*-HSDs have been shown to have opposing functions. 11*β*-HSD2 catalyzes the reversible conversion of active GCs (corticosterone in rats, cortisol in humans) to their inactive 11-keto derivatives (11*β*-dehydrocorticosterone in rats, cortisone in humans) [13]. 11*β*-HSD1 could catalyze both the activation of GCs (where it acts as a reductase), as well the deactivation of GCs (where it acts as a dehydrogenase) [13]. However, in intact cells, the reductase reaction has been shown to be more potent [13]. The importance of 11*β*-HSD activities stems from the fact that GCs are secreted in both active and inactive forms, and it is the tissue-specific activities of 11*β*-HSD that determine the concentration of active GCs within that tissue [14]. Hence any effect of stress or diet on their activities would be an important factor to consider.

Rats are photoperiodic animals that favour darkness over light, thus making them more sensitive to the effects of light [15]. Vanderschuren et al. [16] showed that rats' eyes are conditioned towards dim lighting, between 1–40 lux. Studies conducted by Schlingmann et al. [15] indicated that light intensities as low as 60 lux are sufficient to cause mild distress in rats, while Matsuo and Tsuji [17] showed that prolonged exposure to light intensities above 200 lux caused significant stress. Rats have been shown to have retinal pathologies [18] as well as to undergo many biochemical changes when exposed to light continuously at moderate to high intensities, including fluctuations in the hormones of the HPA axis [19]. This has been shown to alter behaviour (such as feeding) [20], immunological responses [21] and cause disturbances in the function of the female reproductive system [22]. It has even been shown to increase the rate of lipid uptake [23].

This experiment was designed to assess the effects of the typical diet in modern times, as well as the impact of stress which many people face on a day-to-day basis. It also aims to analyse the effects glycyrrhizic acid (GA) will have on any diet/stress-induced effect. Therefore in this experiment, we use a diet high in both carbohydrate and fat. Furthermore, it is very apparent that the intensity of stress faced by different people varies. On the whole when assessing an average person's daily lifestyle pattern, it would seem appropriate to say that in general, the intensity of stress faced is moderate—not extremely stressful but not without stress either. Moreover, many people have to cope with these moderate forms of stress, such as work-related stress, almost constantly in their day-to-day lives. Thus to simulate this pattern, the stress model for this experiment used a moderate and continuous form of stress, that is, moderate intensity lighting of 300–400 lux for the entire duration of the experiment. 

Previous research done in our laboratory has indicated numerous benefits stemming from GA consumption. Improvements in glucose concentrations, insulin concentrations, insulin sensitivities, lipid profiles, 11*β*-HSD activities, and expression of genes involved in lipid metabolism and energy balance have been shown in rats fed on normal, high-fat, and high-sucrose diets separately [24, 25]. These results have shown that it is possible to reap the benefits of GA in doses small enough to avoid the side effect of hypertension, at least on the short term.

The aims of this experiment were to determine the effects of moderate-intensity stress and GA on the following:

- systolic blood pressure,
- serum epinephrine (catecholamines),
- serum corticosterone (glucocorticoids),
- serum glucose, insulin and homeostasis model assessment of insulin resistance index (HOMA-IR),
- 11*β*-HSD 1 and 2 activities in rats fed on a combination diet of high fat and high sucrose.

The null hypothesis for this experiment was that stress or GA will not have any significant effect on any of the aforementioned parameters.

## 2. Materials and Methods

### 2.1. Treatment of Rats and Sample Collection

#### 2.1.1. Animal Ethics

Approval to use rats for the purpose of this experiment has been obtained from the Monash University School of Biomedical Science Animal Ethics Committee (approval no. MARP/2012/043), according to the 2004 Australian Code of Practice for the Care and Use of Animals for Scientific Purposes and Monash University Animal Welfare Committee Guidelines and Policies (Prevention of Cruelty to Animals Act 1986).

#### 2.1.2. Animal Preparation

Eighteen male Sprague Dawley (SD) rats (*Rattus norvegicus*) of approximately 180 g–220 g (~7 weeks old) were purchased from the animal breeding facility of Monash University Sunway Campus. SD rats were chosen as experimental models due to their wide and common use. They are also known to be calmer and therefore offer ease of handling. Only male rats will be used to remove any confounding factors due to cyclic changes in hormones. The rats were housed individually in polystyrene cages of approximately 35 × 25 × 20 cm which contained paper shreddings for bedding. They were acclimatized for 10 days before beginning the experimental treatment in order to get them accustomed to the blood pressure measurement procedure. This was done by restraining the rats using plastic restrainers, covering them using a dark box (which will be used to help calm them), inserting the tail cuff and pulse transducer onto their tails (which will be done for measurement of blood pressure), and subsequently shining a lamp light on their tails (which will be used to dilate their blood vessels).

#### 2.1.3. Experimental Design

The total duration of the experiment was 28 days. Just before treatment, the initial weights of each of the 18 rats were measured before dividing them randomly into three groups, groups A, B, and C. During the course of the experiment, the three groups were fed diet pellets high in both fat and sucrose. The pellets contained 30% animal fat (ghee) and 30% sugar (by weight) mixed together with normal rat chow (Gold Coin, Malaysia). Each rat was given 25 g of the pellets *ad libitum* each day. The amount of food consumed and the weight of each rat were monitored daily.

*Blood Pressure Measurement*. Tail cuff systolic blood pressure readings were also obtained twice a week for each rat across all three groups, using the ADinstruments ML125 NIBP (noninvasive blood pressure) Controller. The rats were restrained and then placed in a dark box, after which a lamp containing a 60 W bulb was shone on their tails for 30 minutes before measuring the blood pressure.

*Stress Conditions*. Group A rats were kept in a room subjected to a 12-hour light-dark cycle (6 am/6 pm) and given tap water to drink. Rats belonging to Groups B and C, however, were kept in a different room and subjected to stress by exposing them to light of an approximate intensity of 300–400 lux (at cage level) for the entire duration of the experiment. All rooms were maintained at 23°C throughout the experimental duration.

*Glycyrrhizic Acid Intake*. While rats in groups A and B were given tap water to drink, those in group C were given water containing 100 mg per kg bodyweight of Glycyrrhizic acid (GA). The GA mixed in water was freshly prepared each week depending on the group C rats' body weights and water consumption. Water bottles were topped to 250 mL every week, and consumption was monitored daily.

#### 2.1.4. Collection of Sample Tissues

On the 28th day all 18 rats were fasted for 12 hours and then anaesthetised prior to dissection using ketamine and xylenol, following the animal ethics guidelines of the institution. The procedure began between 10.00 am and 11.00 am.

Using a 5 mL syringe with a 22 G needle, blood was drawn from the apex of the heart. A microcentrifuge tube containing 0.5 g ethylenediaminetetetraaceticacid (EDTA) and sodium fluoride (NaF) (in a ratio of 1 : 2 w/w) was used to store approximately five drops of blood. EDTA was used to prevent blood clotting, while NaF was used to inhibit glycolysis. The rest of the blood was collected in a sterile falcon tube and left to stand for 20 minutes to allow it to clot. The clotted blood was then sent for centrifugation at 12,000 ×g for 10 minutes at 4°C. The resulting supernatant was then transferred to microcentrifuge tubes and stored at −80°C until needed for measurement of insulin, epinephrine, and corticosterone concentrations.

The six tissues of interest, namely, the subcutaneous and visceral adipose tissue (SAT and VAT), liver, kidney, abdominal muscle (AM), and quadriceps femoris (QF), were harvested and weighed after dissection. Tissues to be used for 11*β*-HSD measurement, that is, the liver, kidney, SAT, VAT, QF, and AM were stored in falcon tubes containing Krebs-Ringer bicarbonate (KRB) buffer. 

### 2.2. Biochemical Analysis

#### 2.2.1. Determination of Blood Glucose Concentration

Trinder's glucose oxidase method was used to determine fasting blood glucose concentration. 100 *μ*L of the whole blood from each rat was separately added to 900 *μ*L of protein precipitant in a microcentrifuge tube. 100 *μ*L of a blank and series of standard ranging from 5 mmol/L to 20 mmol/L were prepared and added with 900 *μ*L of protein precipitant in microcentrifuge tubes. The solution was mixed and centrifuged at 3000 rpm for 15 minutes. Two-hundred and fifty *μ*L of supernatant was aliquoted and added into tubes wrapped with aluminium foil. 750 *μ*L of colour reagent was added into the tube. The solution was mixed and incubated at 37°C for 20 minutes. 200 *μ*L of the solution was then transferred into a microtitre plate. Duplicates were performed for each tube. The microtitre plate was read at 515 nm using Bio TEK Powerwave XS Microplate Scanning Spectrophotometer. Quantification of blood glucose concentrations was performed based on a standard curve. 

#### 2.2.2. Determination of Serum Insulin Concentrations and Homeostasis Model Assessment of Insulin Resistance Index (HOMA-IR)

Serum insulin concentrations were measured using the rat/mouse insulin enzyme-linked immunosorbent assay (ELISA) kit (Millipore, USA). The procedure specified in the manufacturer's manual for the kit was followed. A 96-well microtitre plate was used to conduct the analysis. 

The HOMA-IR index gives an estimation of the degree of insulin resistance of the rats. The higher the value for HOMA-IR, the lower the overall tissue sensitivity towards insulin. The equation used for its calculation is given below:

(1) HOMA-IR =fasting  plasma  glucose  (mmol/L)×fasting  serum  insulin  (ng/mL)22.5.

### 2.3. Serum Epinephrine

Serum epinephrine concentrations were determined using rat epinephrine/adrenaline (EPI) ELISA kit (Cusabio, China). The procedure specified in the manufacturer's manual for the kit was followed. A 96-well microtitre plate was used to conduct the analysis.

### 2.4. Serum Corticosterone

Serum corticosterone concentrations were determined using corticosterone enzyme immunoassay (EIA) kit (Cayman's Chemical Company, USA). The procedure specified in the manufacturer's manual for the kit was followed. A 96-well microtitre plate was used to conduct the analysis.

### 2.5. Determination of 11*β*-HSD 1 and 2 Activities

Prior to the dissection date, Krebs-Ringer bicarbonate buffer (KRB) and 11*β*-HSD 1 and 2 substrate mixtures were prepared. The substrate mixture was aliquoted into microcentrifuge tubes in a volume of 100 *μ*L and these, were stored at 4°C along with the KRB buffer. Throughout the experiment, all reagents, homogenates, and supernatant were kept on ice and all procedures were done at 4°C to prevent proteolysis and denaturation of enzyme, unless stated otherwise.

#### 2.5.1. Homogenization and Treatment of Samples

Tissues collected were weighed, minced into small pieces (to ease homogenization), and placed into 15 mL falcon tubes. For every 1 g of tissue, 2 mL of KRB buffer was added into each tube. The tissues were homogenized using Heidolph DIAX 900 rotor stator homogenizer. After homogenizing each tissue, the homogenizer stator was cleaned with 70% ethanol, distilled water, and KRB buffer, respectively. The homogenates were then centrifuged using the Hettich Zentrifugen Universal 32R centrifuge set at 14,000 ×g for 20 minutes at 4°C.

#### 2.5.2. Protein Concentration Determination

Protein concentrations in tissue homogenates were determined using the modified Lowry's method.

#### 2.5.3. Production of the 11*β*-HSD 1 and 2 Sample Mixtures

According to the standard curve, the homogenate supernatants of each tissue containing 10 mg of tissue protein were separated and incubated with the substrate mixture for both 11*β*-HSD 1 and 2. All six tissues were used to measure 11*β*-HSD 1 activities, whereas the skeletal muscle, QF and AM, were not used for 11*β*-HSD 2 activities. KRB buffer was then used to top up the reaction mixtures to 500 *μ*L, giving a final concentration of 0.35 mmol/L NADP^+^ and NAD^+^ for 11*β*-HSD 1 and 2, respectively, 1 mmol/L corticosterone, 0.2% glucose, 0.2% BSA, and 5% ethanol.

The reaction mixtures were then incubated for 75 minutes at 37°C in a Memmert water bath for one hour. Following this, they were immediately placed into a −20°C freezer and stored there until required.

#### 2.5.4. Glucocorticoid Extraction

The reaction mixtures were removed from −20°C, and 800 *μ*L of ethyl acetate was pipetted into each microcentrifuge tube. Tubes were then horizontally placed on a rotating Protech orbital shaker at 100 rpm for 30 minutes at room temperature. The samples were then centrifuged using the Eppendorf microcentrifuge 5415R at 16000 ×g at room temperature for 10 minutes.

The upper (organic) layer of the centrifuged samples was separated and added to a new microcentrifuge tube, while the lower (aqueous) layer and sediments were thrown. The ethyl acetate in the organic layer was evaporated using flowing nitrogen gas, and dried content was stored at −20°C.

#### 2.5.5. Quantification of Glucocorticoids Using High-Performance Liquid Chromatography (HPLC)

Reverse-phase chromatography was used to separate corticosterone (substrate) from 11-dehydrocorticosterone (product). 300 *μ*L of HPLC mobile phase (20% methanol, 30% acetonitrile, and 50% water v/v) was added to each microcentrifuge tube containing the dried GC content. A series of corticosterone standards were used to make a standard curve prior to quantification of sample GCs. A 100 *μ*L Hamilton HPLC syringe was used to inject 30 *μ*L of standard/sample into a 20 *μ*L sample loop of the Perkin Elmer 200 Liquid Chromatography pump. A Waters Symmetry C18 column with a dimension of 3.9 mm (diameter) × 150 mm (length) was used for the separation of the sample GCs. A linear methanol-acetonitrile-water gradient from 10 : 15 : 75 (v/v) to 20 : 30 : 50 (v/v) in the first five minutes was employed to separate the GCs. Isocratic elution then followed for the next ten minutes. The constant flow rate of the mobile phase was set at 1.00 mL/min. Spectrophotometric absorbance was measured at 254 nm using a Perkin Elmer Series 200 Diode Array Detector. The concentration of corticosterone formed was calculated using the standard curve. 

Enzyme activities were expressed in Units in which 1U is defined as one nanomole (nmol) of substrate (corticosterone) converted to product (11-dehydrocorticosterone) per 10mg of tissue protein used per 75minutes of incubation at 37°C, which is similar to that followed by Chandramouli et al. [24].

### 2.6. Data Analysis

The results obtained were statistically analysed using the Statistical Package for Social Sciences (SPSS) version 16 software for windows. Distribution was assessed using the Shapiro-Wilk test. Data with parametric distribution (all parameters except 11*β*-HSD activities) were analysed using one-way analysis of variance (ANOVA) and Tukey test (which compares means). Nonparametric distributions (11*β*-HSD activities between groups in each tissue) were measured using the Mann-Whitney *U*-test (which compares medians). A statistically significant result across all analyses was denoted by a *P* value equal to or less than 0.05 (*P* ≤ 0.05). 

## 3. Results and Discussion

### 3.1. Serum Epinephrine and Corticosterone

The primary purpose of measuring serum epinephrine, a catecholamine, and serum corticosterone, an inactive glucocorticoid (GC), was to assess the intensity of the stress response at the end of the treatment period of four weeks. Both substances are secreted as part of the stress response [4]. The half-life of corticosterone is approximately 2 minutes [26], while for GCs, it ranges from 70–90 minutes [27]. This tells us that epinephrine and corticosterone will provide evidence for the stress response only on the short term, as both substances will be cleared from the circulation within a few hours of secretion. It must be noted that the adrenal cortex secretes a mixture of both active and inactive GCs in response to stress, and therefore increases in both active or inactive GCs will provide an indication of the stress response [14], hence validating the use of corticosterone for this purpose.

**Figure 1:**
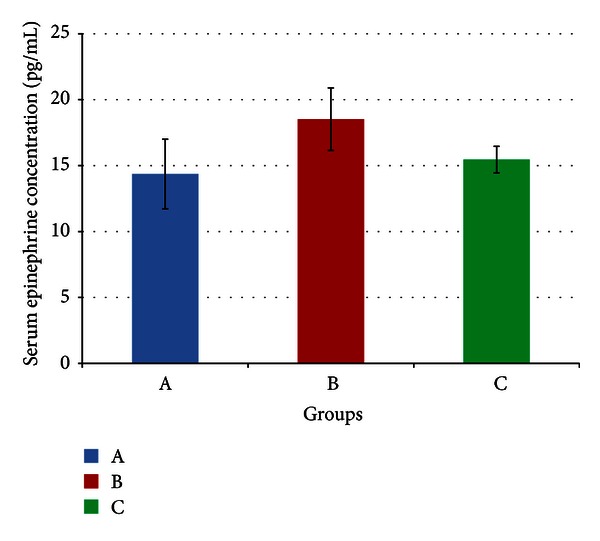
Mean serum epinephrine concentrations of rats in groups A, B, and C at the end of the four-week treatment period. Group A: control (HSD/HFD); group B: stress + HSD/HFD; group C: stress + GA + HSD/HFD. Abbreviations: GA: glycyrrhizic acid, HSD: high-sucrose diet, and HFD: high-fat diet.

Figure 1 indicates no significant difference in mean serum epinephrine concentrations at the end of the four-week treatment period between the three groups (*P* > 0.05). The values for mean serum epinephrine were 14.36 (±2.64), 18.52 (±2.37), and 15.46 (±1.01) pg/mL for rats in group A, B, and C respectively. 

**Figure 2:**
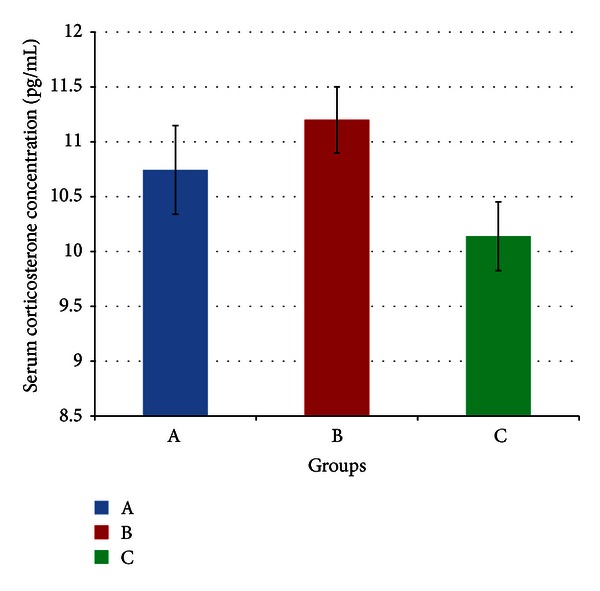
Mean serum corticosterone concentrations of rats in groups A, B, and C at the end of the four-week treatment period. group A: control (HSD/HFD); group B: stress + HSD/HFD; group C: stress + GA + HSD/HFD. Abbreviations: GA: glycyrrhizic acid, HSD: high-sucrose diet, and HFD: high-fat diet.

Figure 2 meanwhile indicates no significant difference in mean serum corticosterone concentrations between the three groups (*P* > 0.05). The mean values for serum corticosterone for Groups A, B, and C were 10.74 (±0.40), 11.2 (±0.30), and 10.14 (±0.31) respectively. 

The lack of a significant difference in epinephrine and corticosterone concentrations between stressed rats and control rats observed in this experiment indicates that the rats had adapted (or habituated) to the stressor at the time the experiment was completed. Studies have shown that repeated administration of low to moderate-intensity stressors, light in this case, leads to adaptation. As a result of adaptation, the “stressful nature” of the stressor, or the stress intensity felt by the animal, gradually declines, and therefore so does the stress response, resulting in GC and catecholamine concentrations diminishing towards normal physiological levels [4]. The fact that the rats did respond to the stressor at some point can be understood when analysing systolic blood pressure and 11*β*-hydroxysteroid dehydrogenase (11*β*-HSD) activities; the results of both these parameters will be analysed subsequently.

Glycyrrhizic acid (GA) has been shown to increase the rate at which adaptation to stress occurs [28, 29], and therefore would reduce circulating GC concentrations in stressed animals faster if the animal is able to adapt to the stressor. However, as mentioned previously, adaptation had already taken place even in the stressed animals, so this effect of GA would no longer be observable. Hence it may be expected that there would be an increased concentration of inactive GCs, such as corticosterone, because GA inhibits 11*β*-HSD 1 activities in tissues such as the liver, kidney, and adipose tissue. Still, it must be noted that this inhibition occurs in a tissue-specific manner [30] and hence may not significantly affect circulating fractions. This would explain why corticosterone concentrations were not significantly increased in GA-fed stressed (GFS) rats compared to controls (Figure 2). GA is not known to directly affect catecholamine levels, and this is indicated in Figure 1 by the lack of a significant difference in epinephrine in GFS rats compared to controls. On the contrary, catecholamines have been proposed to oppose the inhibition of 11*β*-HSD by GA during stress, thereby reducing its efficiency [31]. 

If both catecholamines and corticosterone concentrations were measured earlier in the experiment (before adaptation had fully taken place), it *may* have been possible to see significant increases with stressed rats compared to controls (due to the stress response) and significant decreases with GFS rats compared to stressed rats (due to an enhanced rate of adaptation) in terms of both substances. 

### 3.2. Systolic Blood Pressure

Systolic levels are a good indicator of blood pressure even without the diastolic readings [32]. Hence in this experiment, the systolic levels alone are considered to be sufficient in the analysis of the blood pressure readings between the three groups. The analysis of systolic blood pressure (SBP) was undertaken to understand how the applied stress levels affected the rats across the timeline of the experiment, as stress is known to increase blood pressure [33]. Therefore SBP could give an idea of how stress affected the rats on a real-time basis. SBP was also analysed to investigate reported side effects of GA. Studies have indicated that GA may elicit mineralocorticoid-like effects such as increased sodium retention and hyperkalaemia, by increasing the proportion of active GCs within the kidney. As a result, hypertension may occur due to increased osmotic passage of water due to high sodium concentrations in the blood [34]. 

**Figure 3:**
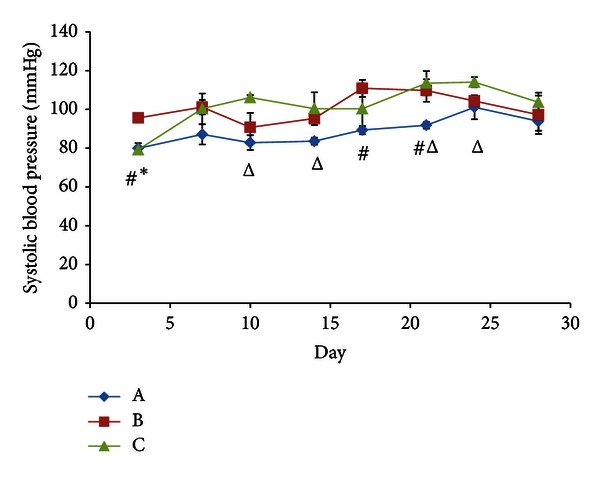
Mean systolic blood pressure of rats in each group across the four-week period, measured twice a week for each rat. ^#^depicts a significant difference between Group A and B, ^Δ^depicts a significant difference between groups A and C, and *indicates a significant difference between groups B and C (significant difference indicated by *P* < 0.05). Group A: control (HSD/HFD); group B: stress + HSD/HFD; group C: stress + GA + HSD/HFD. Abbreviations: GA: glycyrrhizic acid, HSD: high-sucrose diet, and HFD: high-fat diet.

Figure 3 indicates an overall increase in SBP in stressed rats up to day 17 compared to control rats, followed by a period of stasis until day 21 and a gradual decrease thereafter, reaching control levels at day 24. It is apparent that before day 17, the SBP levels do fluctuate slightly, but this could be accounted to minor variations that typically occur when measuring blood pressure using noninvasive blood pressure technologies [35]. 

Before considering stress response-related mechanisms to explain this observation, let us consider the possibility that the effects of continuous light on circadian rhythms may have been responsible for this observation. Many studies have indicated that exposure of animals to continuous light (especially in the case of nocturnal animals, such as rats) diminishes or abolishes certain circadian diurnal rhythms [36–39], which includes the lowering of blood pressure at night [40]. Light is the primary entrainment factor for the mammalian circadian pacemaker—the suprachiasmatic nucleus (SCN) [40]. Furthermore, the diurnal variation in the release of the hormone melatonin by the pineal gland is also regulated by light exposure—its secretion increases during sleep at night [41]. Both the SCN and melatonin have been shown to play a part in blood pressure regulation, and interferences in their normal activities, especially in the case of lowered melatonin secretion due to continuous light exposure, could cause an increase in blood pressure [42]. However, it is unlikely that the effects of light on the SCN and melatonin are the major cause of the observed results of SBP. If it were, then it would be expected that there would be no decrease in pressure at the latter part of the experiment, since the effects of continuous light exposure on the SCN and melatonin have been shown to be maintained until the stimulus is removed [40]. Nevertheless, it is possible that this may have played a small part in the observed initial increase in SBP.

An experiment conducted by Gallara et al. [43] indicated that light can increase mRNA levels of enzymes involved in catecholamine synthesis such as tyrosine hydroxylase (TH), dopamine *β*-hydroxylase (DBH), and phenylethanolamine N-methyltransferase (PNMT), outside the stress response. The resulting increase in catecholamines could have also played a part in the observed increase in SBP at the early points of the experiment, primarily via the increased stimulation of the SNS. 

However, the stress response is a more likely explanation for the observed results. The initial rise in SBP when considering the stress response would be primarily due to the increase in circulating catecholamines caused by activation of the hypothalamic-pituitary adrenal (HPA) axis and stimulation by the sympathetic nervous system (SNS). It is well known that exposure to novel, stressful environments increases catecholamine-mediated SNS stimulation of the cardiovascular system, thereby increasing heart rate and blood pressure [44]. SPB is increased by constricting muscle vasculature and by increasing peripheral vascular resistance [45]. GCs secreted during stress enhance the synthesis and secretion of catecholamines, thereby further enhancing these effects [46].

Following day 17, however, Figure 3 indicates a gradual drop in SBP, and it could be inferred therefore that adaptation to the stressor started around this time. By the end of the experiment, Figures 1 and 2 indicate no significant difference in both epinephrine and corticosterone respectively between control and stressed rats, indicating that the stress response has been minimized at this point. For this reason, at day 28, SBP in stressed rats had reached control levels.

Figure 3 also indicates that at days 10, 14, and 24, GFS rats had a significantly higher SBP than control rats while stressed rats, did not. This indicates that GA feeding did cause a greater impact on SBP than stress at these points. However, the lack of a significant difference between the GFS and stressed rats at any point of the experiment except at the very beginning (day 3, at which mean SBP in stressed rats was significantly higher) indicates that the impact was not substantial. Furthermore, GFS rats had a similar decrease in SBP to control levels towards the end of the treatment period, indicating further that stress, and not GA or its metabolic products, was the primary cause of the observed fluctuations in GFS rats. This corresponds to previous results obtained in our laboratory by Eu et al. [25], which showed no GA-induced increase in blood pressure in rats fed on high-fat diets, while Chandramouli et al. [24] showed the same result in rats fed on high-sucrose diets within the administered dosage of GA. This provides strong evidence for the likelihood that the observed changes in blood pressure were due to factors other than GA, in this case, stress. The likely cause for the insignificant differences in SBP when comparing GFS rats with stressed rats would be an inadequate dosage and/or treatment time.

It is important to note here that SBP cannot be used to assess the capability of GA to enhance adaptation as GA increases SBP as one of its side effects. Although it was stated that GA was unlikely to have caused a significant increase in SBP beyond that of stressed rats in this experiment, it is still preferable to use other factors such as GC concentrations, catecholamine concentrations, and 11*β*-HSD activities when analysing the effects of GA on adaptation.

### 3.3. Blood Glucose, Serum Insulin, and Homeostasis Model Assessment of Insulin Resistance Index

As functional antagonists of insulin, the stress hormone GCs are known to decrease insulin sensitivity as well as increase blood glucose via a variety of pathways including (a) upregulating the transcription of rate limiting enzymes of gluconeogenesis such as phosphoenolpyruvate carboxykinase (PEPCK), and glucose-6-phosphatase (G6P), (b) increasing proteolysis and lipolysis, thereby increasing amino acids and glycerol levels which can act as substrates for gluconeogenesis, (c) decreasing pancreatic insulin secretion, and (d) inhibiting glucose transporter-4 (GLUT-4) which is involved in the uptake of glucose into peripheral tissues [47–49]. Catecholamines meanwhile can also increase blood glucose concentrations by stimulating the secretion of cortisol and glucagon, as well as by enhancing metabolic rate, glycogenolysis, and gluconeogenesis [50]. The resulting GC- and catecholamine-induced increase in blood glucose and decrease in insulin and tissue insulin sensitivities would help meet the increased energy demands of the stressor. However, chronic maladaptive exposure to stress could lead to a variety of metabolic breakdowns due to continuously high concentrations of GCs and catecholamines.

**Figure 4:**
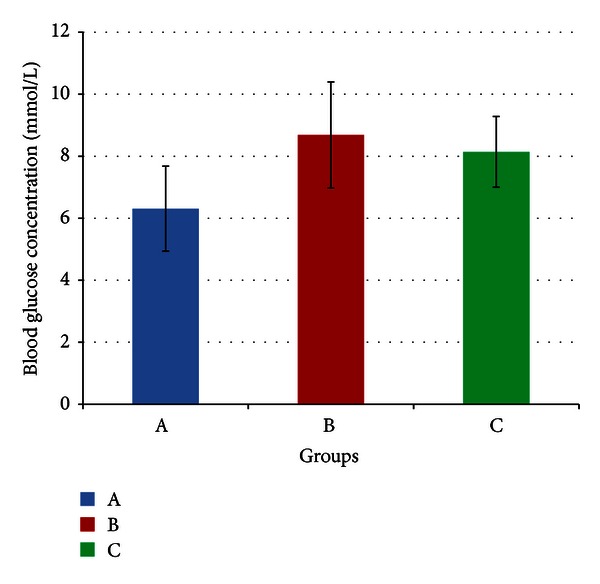
Mean blood glucose concentrations of rats in groups A, B, and C at the end of the four-week treatment period. Group A: control (HSD/HFD); group B: stress + HSD/HFD; group C: stress + GA + HSD/HFD. Abbreviations: GA: glycyrrhizic acid, HSD: high-sucrose diet, and HFD: high-fat diet.

Figure 4 indicates no significant difference in mean blood glucose concentrations between the three groups (*P* > 0.05) at the end of the four-week period. Mean blood glucose concentrations were 6.31 (±1.37), 8.69 (±1.71), and 8.14 (±1.14) mmol/L for groups A, B and C respectively. 

**Figure 5:**
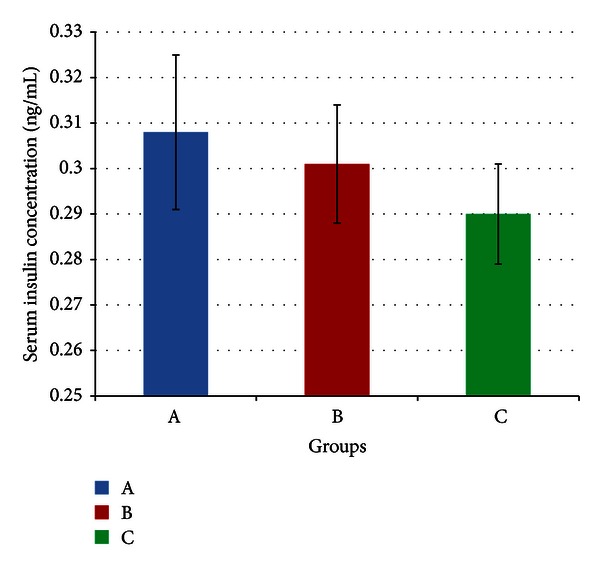
Mean serum insulin concentrations of rats in groups A, B, and C at the end of the four-week treatment period. Group A: control (HSD/HFD); group B: stress + HSD/HFD; group C: stress + GA + HSD/HFD. Abbreviations: GA: glycyrrhizic acid, HSD: high-sucrose diet, and HFD: high-fat diet.

Meanwhile, Figure 5 indicates a mean serum insulin concentration of 0.308 (±0.017), 0.301 (±0.013) and 0.290 (±0.011) ng/mL for groups A, B, and C, respectively. There was no significant difference in insulin concentrations between the three groups (*P* > 0.05) at the end of four weeks.

**Figure 6:**
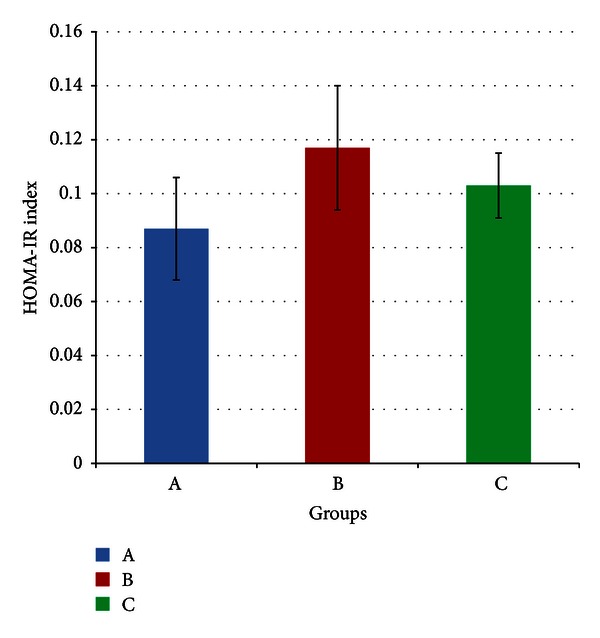
Mean HOMA-IR index of rats in groups A, B, and C at the end of the four-week treatment period. Group A: control (HSD/HFD); group B: stress + HSD/HFD; group C: stress + GA + HSD/HFD. Abbreviations: GA: glycyrrhizic acid, HSD: high-sucrose diet, and HFD: high-fat diet.

The HOMA-IR index for each group is shown in Figure 6. It shows that no significant difference was obtained between the groups (*P* > 0.05) at the end of four weeks. The HOMA-IR index for groups A, B, and C were 0.087 (±0.019), 0.117 (±0.023), and 0.103 (±0.012), respectively. 

As predicted by SBP, the lack of any significant difference in the parameters of glucose metabolism measured in this experiment is likely because the stressed rats had adapted to the stressor at the end of four weeks. Hence the GC and catecholamine concentrations, which were likely to have been elevated before adaptation, had reached control levels by the end of the experiment as shown in Figures 1 and 2. As such, stress response-induced elevations in glucose and reduction in insulin concentrations and tissue insulin sensitivities were no longer present. This shows that there were no long-lasting effects on glucose metabolism and insulin sensitivities caused by the type and intensity of the stressor used in this experiment.

That said, an experiment conducted by Niijima et al. [51] indicated that exposure to light caused an increase in glucose production in rats outside of the HPA axis and stress response. This was accounted to an increase in sympathetic outflow from the SCN of the hypothalamus towards visceral organs, which was shown to facilitate glucagon secretion and repress insulin secretion. However, acute exposure to extremely high light intensities, up to 2000 lux, was used in that experiment and therefore cannot be compared to the present experiment. 

Previous experiments conducted in our laboratory have indicated that GA significantly improved blood glucose and insulin sensitivities in rats fed on both high-fat [25] and high-sucrose diets [24]. With high-sucrose diets, GA was shown to inhibit 11*β*-HSD 1 activities across all tissues studied and therefore reduce active GC concentrations within those tissues [24]. Furthermore, Alberts et al. [52] used 11*β*-HSD 1 gene knockout mice to demonstrate that inhibition of 11*β*-HSD 1 could reduce blood glucose without significant risk of hypoglycaemia. Their experiment further indicated that activation of peroxisome proliferator-activated receptor gamma (PPAR*γ*) (which is also an action of GA) along with 11*β*-HSD 1 inhibition leads to decreased expression of gluconeogenic enzymes. This has been proven in our laboratory by Chandramouli et al. [24] who showed significant GA-induced decreases in PEPCK and G6Pase activities in rats fed on high-sucrose diets. This is a very important characteristic of GA as it has been shown that up to 90% of the glucose released from the liver in T2DM is due to accelerated gluconeogenesis [52]. Lowered active GCs would also result in increased glucose uptake by GLUT-4 into peripheral tissues as well as improved insulin sensitivities, and these have also been shown to be mediated by PPAR*γ* activation and 11*β*-HSD 1 inhibition [53]. Reduced insulin secretion was accounted to upregulation of glucose-sensing proteins within the pancreatic *β*cells via PPAR*γ* activation [54]. As insulin secretion occurs in response to circulating glucose concentrations [55], this could result in an increase in the glucose threshold for insulin, resulting in lower levels of secretion [24]. 

As mentioned before, there were no significant differences in blood glucose, serum insulin, and insulin sensitivities between stressed rats and control rats by week four of the experiment. Since stress had not brought about any changes, it may be expected that GA would improve blood glucose and insulin sensitivities compared to controls. This is because the controls were also fed on a diet high in both fat and sucrose and previous experiments (as mentioned previously) have shown that GA can counter the adverse effects of these diets on glucose metabolism. However, according to Figures 4, 5 and 6, there was no significant difference between GA and control or stressed rats with regard to blood glucose serum insulin and insulin sensitivities. This is likely to be due to the effects of stress prior to adaptation. As shown in Figure 3 by SBP, adaptation likely began around day 17–21, and before that, there may have been stress-induced aberrations in glucose metabolism. If this were true, then for a greater part of the experiment, the beneficial effects of GA with regard to glucose metabolism would go primarily into negating these effects of stress. By the time adaptation was complete, there may have been an insufficient duration for GA to improve these factors beyond that of control levels. If this were proven true, it would also indicate a potential mechanism by which GA enhances adaptation to stress. If GA were to reduce the aberrances in glucose metabolism caused by stress, then following adaptation, glucose levels and insulin sensitivities would likely return to control levels faster in GA-treated rats. 

Another possibility for the observed lack of significant GA-induced effects is that the increase in catecholamines that would have occurred prior to adaptation would have opposed the ability of GA to inhibit 11*β*-HSD, as shown by Farihah et al. [31]. This would diminish the overall efficiency of GA in reducing the proportion of active GCs, and therefore, reduce its ability to improve glucose and insulin levels, as well as insulin sensitivities. There may be other factors other than catecholamines involved here too, and this possibility will be discussed subsequently.

### 3.4. 11*β*-Hydroxysteroid Dehydrogenase 1 and Activities

The extent of GC-induced effects within tissues is determined by 11*β*-HSD activities within that tissue. The changes in 11*β*-HSD activities seen in many of the tissues in this experiment provide further evidence, along with SBP, that the stressor indeed did cause changes in the rats exposed to it. 

Burén et al. [56] indicated in their experiment that there is no diurnal variation in 11*β*-HSD 1 activities in liver, fat and muscle tissues. Therefore it is unlikely that light had any effect on 11*β*-HSD 1 in any of these tissues outside the stress response, unless specifically mentioned.

11*β*-HSD 1 and 2 activities between the 3 Groups in the liver, kidney, SAT, VAT, QF, and AM are shown individually in Figures 7–16. 

#### 3.4.1. Liver

As the liver is an important organ with regard to energy metabolism, playing a crucial role in both energy storage and release [57], it is not surprising that it has a high expression of GC receptors [58]. Furthermore, previous experiments conducted in our laboratory indicate that it has the highest activity of the 11*β*-HSD 1 isozyme [24]. 

As mentioned before, GCs stimulate hepatic gluconeogenesis by upregulating the transcription of the PEPCK and G6P genes. GCs also increase hepatic glycogenolysis and prevent glycogen synthesis [59]. As GC receptors are low-affinity receptors [58], a high expression of is are important in order to adequately mobilize energy reserves during situations such as stress. Similarly, it would be expected that 11*β*-HSD 1 activities would increase due to stress, in order to increase concentrations of active GCs. This can be confirmed by assessing an experiment by Altuna et al. [30] who showed that stress induction for just a few hours caused an increase in 11*β*-HSD 1 activities in the liver due to an increase in NADPH, as well as a reduction in pH due to anaerobic glycolysis that occurs during stressful situations. NADPH acts as a cofactor for 11*β*-HSD 1, while a lower pH favours the reductase reaction of 11*β*-HSD 1, both of which will increase the catalytic action of the conversion of inactive GCs to active GCs [60].

However, results from an experiment conducted by Jamieson et al. [61] suggested that GCs gradually repress the activities of 11*β*-HSD 1 in the liver over time. This may be thought of as somewhat of a negative-feedback loop that prevents excessive formation of GCs over time. This repression of 11*β*-HSD 1 activities by GCs is tissue specific, the liver being one of the few in which it has been found to occur [61]. That said, Farihah et al. [62] suggested that it was unlikely that GCs were responsible for modulating the stress-induced effects on 11*β*-HSD.

**Figure 7:**
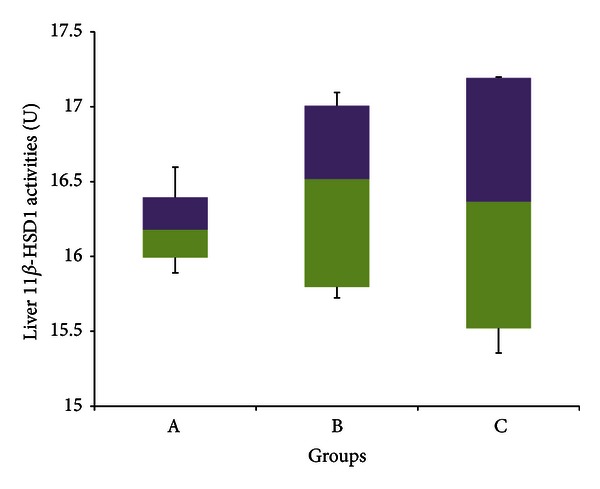
11*β*-HSD 1 activities in the liver of rats in Groups A, B, and C at the end of the four-week treatment period. Group A: control (HSD/HFD); group B: stress + HSD/HFD; group C: stress + GA + HSD/HFD. Abbreviations: GA: glycyrrhizic acid, HSD: high-sucrose diet, and HFD: high-fat diet.

The median hepatic 11*β*-HSD 1 enzyme activities depicted in Figure 7 were 16.178 U (15.891–16.596 U) for group A, 16.516 U (15.724–17.095 U) for group B, and 16.366 U (15.355–17.199 U) for group C. Overall, there was no significant difference between any of the three experimental groups for 11*β*-HSD 1 enzyme activities in the liver (*P* > 0.05 in all cases). 

**Figure 8:**
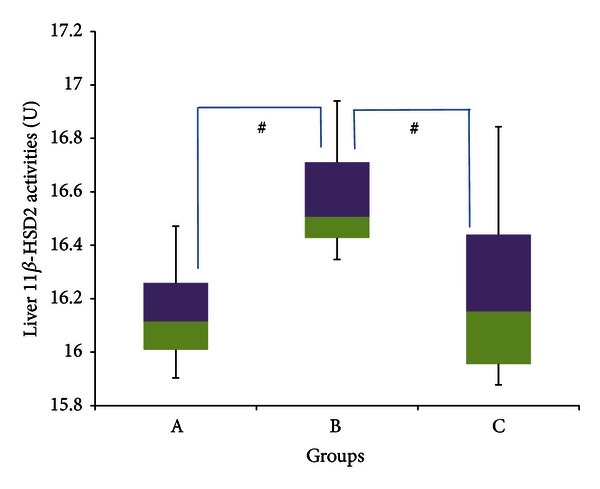
11*β*-HSD 2 activities in the liver of rats in groups A, B, and C at the end of the four-week treatment period. ^#^Significant difference (*P* < 0.05) between denoted groups. Group A: control (HSD/HFD); group B: stress + HSD/HFD; group C: stress + GA + HSD/HFD. Abbreviations: GA: glycyrrhizic acid, HSD: high-sucrose diet, and HFD: high-fat diet.

Figure 8 meanwhile shows hepatic 11*β*-HSD 2 enzyme activities, in which Group B showed significantly greater activities, with a median of 16.506 U (16.347–16.942 U), compared to Group A which had a median enzyme activity of 16.114 U (15.904–16.471 U) and group C which had a median enzyme activity of 16.152 U (15.877–16.844 U) (*P* < 0.05 in both cases). The percentage increase in median enzyme activities in group B compared to group A was ~2%. The percentage decrease in median enzyme activities in Group C compared to group B was also ~2%.

The lack of a significant difference in hepatic 11*β*-HSD 1 activities between stressed and control rats concurs with an experiment conducted by [63], which indicated that mild chronic stress with a high-fat diet caused no significant changes in 11*β*-HSD 1 activities. Although this was accounted to the possibility that a palatable diet could diminish stress effects, there are a number of other possible reasons for the lack of a change in 11*β*-HSD 1 activities in stressed rats. First, as mentioned before, there *may* exist a tissue-specific repression of 11*β*-HSD 1 activities by GCs in the liver, as indicated by Jamieson et al. [61], although Farihah et al. [62] suggested otherwise.

Furthermore, the gradual adaptation of the rats to the stressor as indicated by the fall in SBP towards the latter part of the experiment may also have played a part, as the need for GCs decreases with adaptation [4]. This may possibly explain the significant increase in hepatic 11*β*-HSD 2 activities in stressed rats (Figure 8). Although not produced in the liver directly, it is possible that 11*β*-HSD 2 produced in the epithelial cells [64] and smooth muscle [65] of the surrounding blood vessels (which are found in high density in the region of the liver) may have been upregulated in order to help in bringing GC levels down to normal physiological concentrations. If this was the case, upregulation of 11*β*-HSD 2 in the region of the liver would be especially advantageous as GCs have substantial effects on the liver [59]. However, with the lack of work done on the effects of stress on 11*β*-HSD 2 activity in the hepatic region, especially immediately following adaptation, more work needs to be done in order to provide evidence for this.

Previous research done in our laboratory showed that GA decreased hepatic 11*β*-HSD 1 activities in rats fed on a high-fat diet as well as a high-sucrose diet alone [24]. Since stress had no significant effect on 11*β*-HSD 1 activities, it would be expected therefore that GA would reduce the enzyme activities below control levels, as the controls are fed a combination of a high-fat and high-sucrose diet in this experiment. However, Figure 7 indicates no significant difference in 11*β*-HSD 1 activities between GFS rats and control rats. Repetitive stress has been shown to be able to overcome the inhibition of 11*β*-HSD 1 by GA in the liver and kidney. Farihah et al. [31] suggested that catecholamines secreted during stress were responsible for this, at least in part. However at the point at which the measurement of enzyme activities was made, catecholamine levels had already reached control levels due to adaptation (Figure 1). This suggests that some other factors(s) secreted during the stress response may also be able to block the GA-mediated inhibition of 11*β*-HSD 1 in the liver. Any factor involved would likely be one which production is not as easily diminished following adaptation, and/or has a longer half-life. It is also possible that the effects of the factor(s) concerned on 11*β*-HSD 1 activities are longer lasting compared to catecholamines, so even after the stress response ceases, their influence would still be apparent.

Activities of 11*β*-HSD 2 were significantly reduced in GFS rats compared to stressed rats, while there was no significant difference between GFS rats and controls. This indicates that the increase in 11*β*-HSD 2 activities in the liver caused by stress (or by the adaptation to stress, as proposed before) was substantially countered by GA intake. This indicates that the factor(s) (other than catecholamines) that may be responsible for blocking hepatic 11*β*-HSD 1 inhibition by GA do not have the same effect on 11*β*-HSD 2.

#### 3.4.2. Kidney

**Figure 9:**
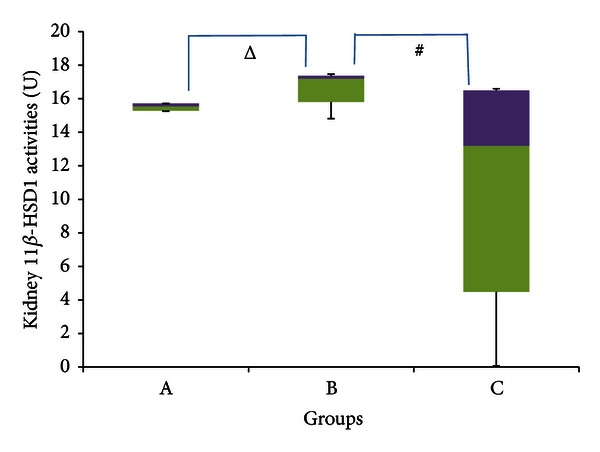
11*β*-HSD 1 activities in the kidney of rats in group A, B, and C at the end of the four-week treatment period. ^#^Significant difference (*P* < 0.05) between denoted groups. ^Δ^Weakly significant difference (*P* = 0.05) between denoted groups. Group A: control (HSD/HFD); group B: stress + HSD/HFD; group C: stress + GA + HSD/HFD. Abbreviations: GA: glycyrrhizic acid, HSD: high-sucrose diet, and HFD: high-fat diet.

According to Figure 9, a significant decrease in renal 11*β*-HSD 1 activities occurred in group C compared to group B (*P* < 0.05), which had medians of 13.181 U (0.068–16.592 U), and 17.195 U (14.814–17.461 U), respectively, indicating a percentage decrease of ~23%. Group B showed greater enzyme activities compared to group A, which had a median enzyme activity of 15.551 U (15.253–15.718 U) but with a relatively weak significance (*P* = 0.05). The percentage increase in group B compared to group A was ~10%. There was no significant difference in median 11*β*-HSD 1 activities between Group A and C (*P* > 0.05).

**Figure 10:**
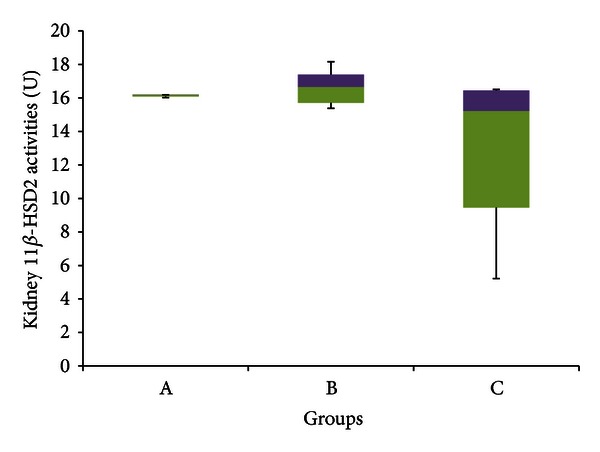
11*β*-HSD 2 activities in the kidney of rats in group A, B and C at the end of the four-week treatment period. Group A: control (HSD/HFD); group B: stress + HSD/HFD; group C: stress + GA + HSD/HFD. Abbreviations: GA: glycyrrhizic acid, HSD: high-sucrose diet, and HFD: high-fat diet.

Figure 10 indicates that there was no significant difference in renal 11*β*-HSD 2 activities between any of the three experimental groups (*P* > 0.05). Group A had a median enzyme activity of 16.186 U (16.028–16.188 U), group B had a median enzyme activity of 16.666 U (15.381–18.165 U), and Group C had a median enzyme activity of 15.221 U (5.220–16.509 U).

GC receptors have been found to be expressed in proximal tubules of the rat kidney [66]. This suggests that rat kidneys have a need for GCs despite being MC receptors rich organs. It is known that although glucose production in the kidney is generally very low, it does have the capacity to increases greatly during times of stress [67] and diabetes [68]. As all rats in this experiment have diet-induced diabetes; the increased need of glucose in the stressed rats probably resulted in the higher activities of 11*β*-HSD 1 in order to increase GC-mediated gluconeogenesis. Quinkler et al. [69] showed that in guinea pigs, there was a significant increase in renal 11*β*-HSD 1 activities when exposed to stress. The weak significance of the increase in 11*β*-HSD 1 activities in the stressed rats in the present experiment could be accounted to adaptation taking place. Hence at the point of measurement, the enzyme activities were likely to be declining. 

Renal 11*β*-HSD 2 converts active GCs to inactive GCs in order to protect against hypermineralocorticoid effects, including hypertension, as the kidney tissue is rich in MC receptors towards which GCs have a high affinity. As such, there is a high expression of 11*β*-HSD 2 in the kidney [34]. 

Inactive GCs have low binding affinity towards corticosteroid-binding globulin and albumin compared to active GCs and are thus more likely to be found in the free form (the biologically active form). Furthermore, inactive GC concentrations do not show diurnal variations [12]. Thus, the circulating inactive GCs could act as a substrate pool which could be activated by 11*β*-HSD 1 in the adipose tissues and the liver. This will allow 11*β*-HSD 1 to maintain or even increase the active GC concentrations in the adipose tissue and liver [70]. Therefore, it is possible to consider that during stress, there should be an increase in 11*β*-HSD 2 activities. However, many studies have shown decreased hepatic 11*β*-HSD 2 activities [71–73] or no significant change in 11*β*-HSD 2 activities [62] when exposed to stress-like conditions. One possibility for this is the proposed inhibition of 11*β*-HSD 2 by ACTH-induced steroids, such as progesterone and its metabolites, and also corticosterone [74, 75], although other research suggests that corticosterone plays no part in the modulation of 11*β*-HSD activities during stress [62]. 

As can be seen in Figure 10, this experiment indicated no significant difference in activities of 11*β*-HSD 2 between stressed rats and control rats, supporting the result obtained by Farihah et al. [62]. The physiological basis for such a decrease or lack of change in 11*β*-HSD 2 is not yet certain. This author postulates that, since gluconeogenesis does occur in the kidney, the repression of 11*β*-HSD 2 will allow for increased active GCs within the immediately surrounding area, thereby enhancing renal gluconeogenesis in order to compensate for the additional glucose demands of stress. The fact that 11*β*-HSD 1 activities increase within the kidney during stress, which would also aid in enhancing renal gluconeogenesis, indicates that this notion may be a possibility. Furthermore, increased 11*β*-HSD 2 activities would suppress increases in blood pressure caused by GCs binding to MC receptors. This would be disadvantageous as higher blood pressure would allow better rates of transport of nutrients and oxygen to the skeletal muscles and brain, indicating another advantage of restricting the activity of renal 11*β*-HSD 2 during stress. 

Figure 9 indicates a significant reduction in 11*β*-HSD 1 activities in GFS rats compared to stressed rats. Here we see that GA has countered the stress-induced increase in 11*β*-HSD 1 activities. Farihah et al. [31], however, indicated that catecholamines restrict the inhibition of renal 11*β*-HSD 1 by GA. Nevertheless following the period of adaptation, the decline in catecholamine levels would have minimized this effect and allow for the GA-induced inhibition of renal 11*β*-HSD 1 to occur. As mentioned before, glucose production in the kidney increases substantially during stress, in order to meet the increased energy demands of the organism. This upsurge may be aided by increased concentrations of active GCs produced by higher 11*β*-HSD 1 activities induced by stress. However, even if increased GC levels are necessary during stress, chronic elevations, especially in organs heavily involved in glucose metabolism, result in a variety of problems which were previously mentioned. These may culminate in type II diabetes mellitus (T2DM) and cardiovascular disease (CVD). Hence the repression of 11*β*-HSD 1 activities by GA would be advantageous as this may be (at least in part) the reason GA enhances adaptation, allowing the organism to return to normal physiological states faster after a period of stress. In fact, the inhibition of renal 11*β*-HSD 1 by GA would make it likely that it also hastened the normalizing of blood glucose and possibly even insulin sensitivities, giving further evidence for the ability of GA to increase the rate of adaptation to stress. 

The experiment indicated no significant difference in the activity of 11*β*-HSD 2 between GFS and stressed rats as well as controls (Figure 10). This agreed with the results of a previous experiment by Farihah et al. [31], who showed that even in the absence of stress, GA may not cause a significant difference in renal 11*β*-HSD 2 activity. If GA indeed does not interfere with renal 11*β*-HSD 2 activities, it would seem advantageous as this would minimize increases in active GCs in the kidney. However, the hypermineralocorticoid effects of GA are well established, and even if it does not inhibit renal 11*β*-HSD 2, it is likely to increase the proportion of active GCs in the kidney in some other indirect manner.

#### 3.4.3. Adipose Tissue

Adipose tissues are specialized for lipid storage, with lipid droplets composing up to 90% of the volume of an adipocyte, and being capable of up to a thousandfold increase when needed [76]. In recent years, the function of adipose tissue has extended to the secretion of adipocytokines such as leptin, TNF-*α*, and interleukin-6 (IL-6) [76], and the VAT plays a more active role in this respect [77]. GCs play a major role in the regulation of metabolism in adipose tissue as well as in the differentiation of preadipocytes to adipocytes [14]. Many studies have shown that GCs increase lipolysis in mature adipocytes by increased transcription and expression of the adipose triglyceride lipase (ATGL) and hormone-sensitive lipase (HSL) [78–81]. That said, conditions in which GCs increase, such as during stress, have been shown to cause increased adiposity via enhanced adipogenesis [82], increased LPL activities [83, 84], increased food intake [85], and so forth. Thus despite its well-known lipolytic effects, GCs could cause adipocyte hypertrophy which has the potential to lead to obesity. Central obesity is an important component of MetS through which other abnormalities could develop and hence is particularly problematic [86]. An experiment conducted by Bujalska et al. [87] pointed out the importance of 11*β*-HSD 1 in the development of these metabolic aberrations, by showing that inhibiting this enzyme prevented adipogenesis in humans. Furthermore, Seckl et al. [88] showed that 11*β*-HSD 1 knockout mice were resistant to many of the metabolic aberrations that occurred within the liver and adipocytes due to stress and high-fat diet. 

**Figure 11:**
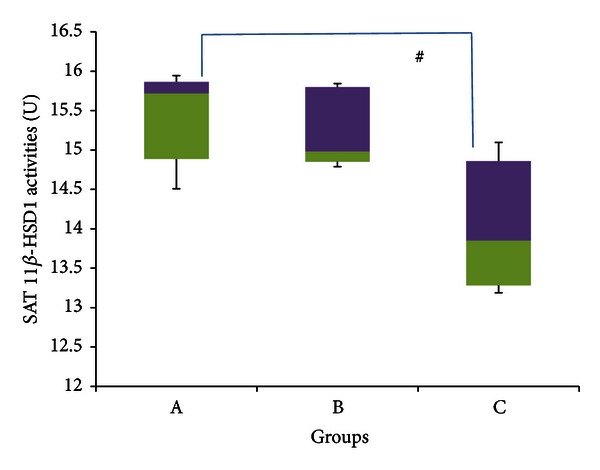
11*β*-HSD 1 activities in the subcutaneous adipose tissue of rats in groups A, B, and C at the end of the four-week treatment period. ^#^Significant difference (*P* < 0.05) between denoted groups. Group A: control (HSD/HFD); group B: stress + HSD/HFD; group C: stress + GA + HSD/HFD. Abbreviations: GA: glycyrrhizic acid, HSD: high-sucrose diet, and HFD: high-fat diet.

Figure 11 indicates significantly lower 11*β*-HSD 1 activities within the SAT of group C rats compared to group A (*P* < 0.05), with medians of 13.851 U (13.189–15.096 U) and 15.722 U (14.509–15.945 U), respectively, indicating a percentage decrease of ~12%. Group B rats meanwhile had a median enzyme activity of 14.982 U (14.791–15.845 U), the value of which was not significantly different from either of the other two groups (*P* > 0.05).

**Figure 12:**
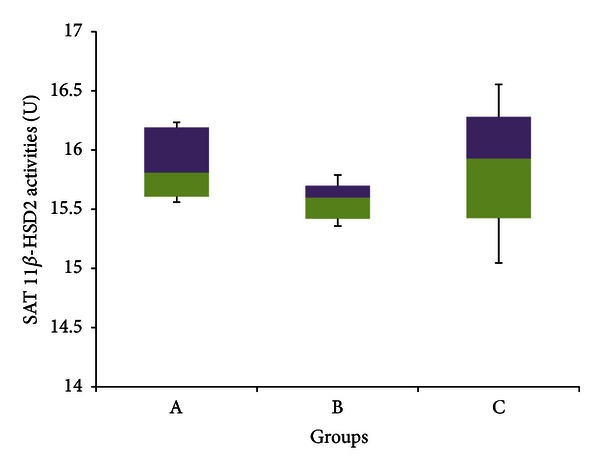
11*β*-HSD 2 activities in the subcutaneous adipose tissue of rats in groups A, B, and C at the end of the four-week treatment period. Group A: control (HSD/HFD); group B: stress + HSD/HFD; group C: stress + GA + HSD/HFD. Abbreviations: GA: glycyrrhizic acid, HSD: high-sucrose diet, and HFD: high-fat diet.

Figure 12 meanwhile shows that there was no significant difference in 11*β*-HSD 2 activities within the SAT between any of the three groups (*P* > 0.05). The median enzyme activities for the three groups were 15.810 U (15.560–16.233 U), 15.598 U (15.358–15.790 U), and 15.928 U (15.047–16.555 U) for groups A, B, and C respectively. 

**Figure 13:**
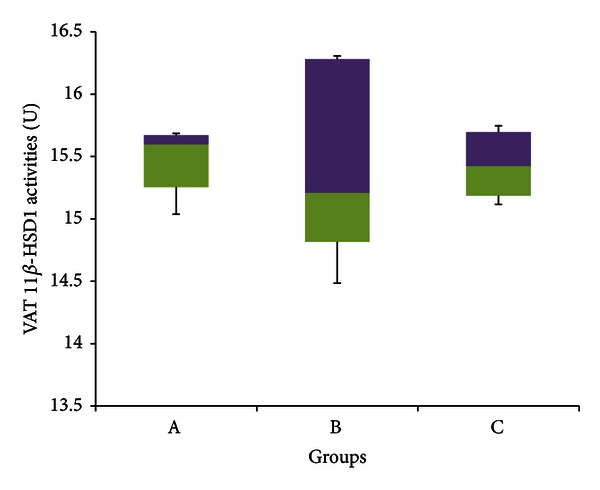
11*β*-HSD 1 activities in the visceral adipose tissue of rats in groups A, B, and C at the end of the four-week treatment period. Group A: control (HSD/HFD); group B: stress + HSD/HFD; group C: stress + GA + HSD/HFD. Abbreviations: GA: glycyrrhizic acid, HSD: high-sucrose diet, and HFD: high-fat diet.

Figure 13 indicates no significant difference in 11*β*-HSD 1 activities in visceral adipose tissue between any of the three groups (*P* > 0.05). The median enzyme activities were 15.597 U (15.039–15.685 U) for group A, 15.209 U (14.485–16.307 U) for group B, and 15.423 U (15.116–15.747 U) for group C.

**Figure 14:**
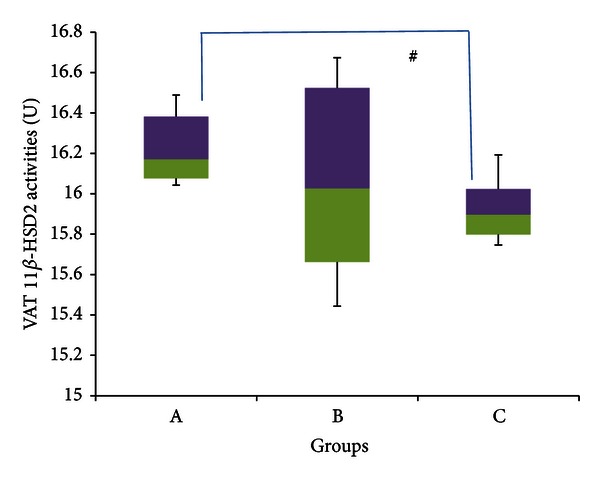
11*β*-HSD 2 activities in the visceral adipose tissue of rats in groups A, B, and C at the end of the four-week treatment period. ^#^Significant difference (*P* < 0.05) between denoted groups. Group A: control (HSD/HFD); group b: Stress + HSD/HFD; group C: stress + GA + HSD/HFD. Abbreviations: GA: glycyrrhizic acid, HSD: high-sucrose diet, and HFD: high-fat diet.

11*β*-HSD 2 activities in visceral adipose tissue, indicated in Figure 14, showed a significant decrease (*P* < 0.05) in group C, with a median of 15.896 U (15.746–16.192 U), compared to group A, which had a median of 16.170 U (16.042–16.489 U) (percentage decrease *≈* 2%). Group B meanwhile had a median of 16.026 U (15.443–16.673 U) and was not significantly different from either of the other two groups (*P* > 0.05). 

Figures 11–14 indicate no significant difference in the activities of 11*β*-HSD 1 and 2 between control and stressed rats in both subcutaneous and visceral adipose tissues. It would be expected, however, that stress would increase 11*β*-HSD 1 activities within adipocytes in order to mobilize their energy stores, which are generally substantial. Previous experiments show that when exposed to stress, 11*β*-HSD 1 activities increase by up to 3.5 times within adipocytes [89]. Furthermore, Engeli et al. [90] revealed that increasing circulating concentrations of cortisol increased 11*β*-HSD 1 expression in isolated human adipocytes. Therefore again, it is likely here that adaptation has offset the increase in 11*β*-HSD activities caused by stress. 

Figure 11 points out that GFS rats had significantly lower median 11*β*-HSD 1 activities compared to control rats in SAT, while Figure 14 indicates that GFS rats had a significantly lower median 11*β*-HSD 2 activities compared to control rats within the VAT. This is slightly different to the results obtained from the liver and kidney, in which GA only countered the increase in 11*β*-HSD activities caused by stress, yet did not lower the activities below control levels. In both these cases, however, enzyme activities were significantly elevated due to stress, so the inhibitory actions of GA would have been sufficient only to counter these increases. In fact, previous research done in our laboratory indicates that GA can significantly reduce high-sucrose diet-induced increases in 11*β*-HSD 1 within the adipose tissue [24]. Hence in the absence of stress-induced increases, it is likely that GA would be able to lower 11*β*-HSD activities beyond control levels as shown in this experiment. The ability of GA to reduce 11*β*-HSD activities in adipose tissue can result in many metabolic improvements, as it would oppose the aforementioned aberrations caused by increased GCs. 

#### 3.4.4. 11*β*-HSD 1 Activities in Skeletal Muscle

Skeletal muscle tissues such as the quadriceps femoris (QF) and abdominal muscle (AM) are major targets for both GCs and insulin actions [49]. In fact, skeletal muscles are considered to be one of the most important sites of insulin-induced uptake of glucose [91]. In the euglycaemic state, the skeletal muscle is responsible for approximately 75% of insulin-mediated glucose uptake, which increases up to 95% in the hyperglycaemic state [91]. However, skeletal muscle also has been shown to express 11*β*-HSD 1 and GC receptors, therefore making it sensitive to conditions in which expression of GC receptors or 11*β*-HSD 1 activities increase [92]. 

An experiment conducted by Morgan et al. [93] indicated that GCs could decrease insulin sensitivity within the skeletal muscle by increasing the phosphorylation of insulin receptor substrate-1 (IRS-1) by serine, which results in decreased affinity for insulin receptor and increased degradation. Their experiment also showed that GCs could reduce the expression of IRS-1, which would also contribute to desensitization of the skeletal muscle tissue towards the actions of insulin. GCs also decrease insulin-stimulated glycogen synthesis by preventing the activation of glycogen synthase by dephosphorylating the enzyme [94], as well as decrease glucose uptake into muscle by inhibiting GLUT-4 [49]. As the skeletal muscle plays a major part in insulin-mediated glucose uptake, reduced insulin sensitivities could be particularly problematic. Furthermore, GCs also increase proteolysis and inhibit protein synthesis in muscle tissue as well as prevent uptake of amino acids into muscle tissue, in order to increase gluconeogenic substrates in the liver [49]. As a result of these GC-mediated effects, mobilization of energy reserves within the muscles takes place, which is an important event during stress. 

**Figure 15:**
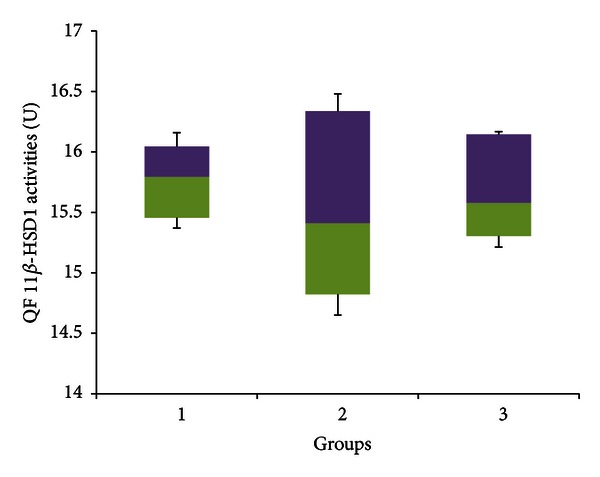
11*β*-HSD 1 activities in the quadriceps femoris of rats in group A, B, and C at the end of the four-week treatment period. Group A: control (HSD/HFD); group B: stress + HSD/HFD; group C: stress + GA + HSD/HFD. Abbreviations: GA: glycyrrhizic acid, HSD: high-sucrose diet, and HFD: high-fat diet.

Figure 15 indicates that there was no significant difference in 11*β*-HSD 1 activities in the quadriceps femoris between any of the experimental groups (*P* > 0.05). The median enzyme activities for groups A, B, and C were 15.794 U (15.370–16.159 U), 15.410 U (14.650–16.480 U) and 15.577 U (15.213–16.167 U), respectively.

**Figure 16:**
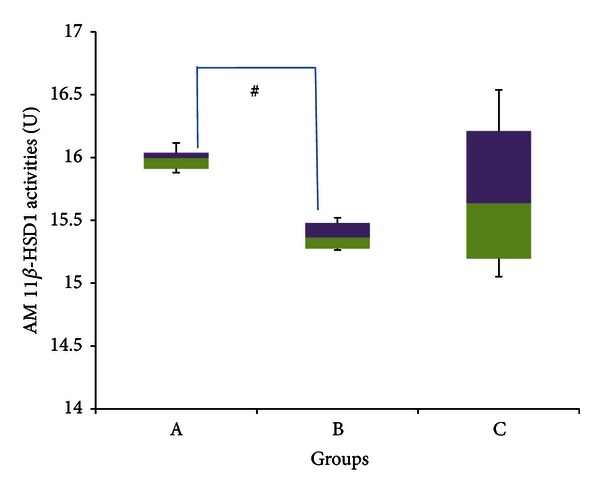
11*β*-HSD 1 activities in abdominal muscle of rats in group A, B, and C at the end of the four-week treatment period. ^#^Significant difference (*P* < 0.05) between denoted groups. Group A: control (HSD/HFD); group B: stress + HSD/HFD; group C: stress + GA + HSD/HFD. Abbreviations: GA: glycyrrhizic acid, HSD: high-sucrose diet, and HFD: high-fat diet.

Figure 16, which depicts median 11*β*-HSD 1 activities in the abdominal muscle, indicates a significant decrease (*P* < 0.05) in group B, which had a median enzyme activity of 15.363 U (15.262–15.520 U), compared to group A which had a median enzyme activity of 15.995 U (15.880–15.115 U) (~4% decrease). Group C meanwhile had a median enzyme activity of 15.636 U (15.051–15.538 U), and was not significantly different from either of the other two groups (*P* > 0.05).

Many studies have already indicated that stress increases the activities of 11*β*-HSD 1 within skeletal muscle. This has been shown using a variety of stressors, ranging from surgery [95], intense exercise [96], and even natural disasters [97]. Jang et al. [95] also showed that, in his experiment, the increase in skeletal muscle 11*β*-HSD 1 activities caused by stress was not associated with changes in any other factors known to upregulate the enzyme activities in other tissues, indicating that a mechanism specific to skeletal muscles may be involved. The results of the present experiment, indicated in Figures 15 and 16, pointed toward a decrease in 11*β*-HSD 1 activities with stress, although this was not significant in the QF muscle. There may be two reasons for this, which when combined, would account for the observed decrease. Firstly, as already discussed, adaptation had taken place by the end of the four weeks, at which time many of the physiological and biochemical changes associated with stress were no longer present (as shown in Figures 1, 2, and 3). Hence it would be expected that,at this point, there would be no significant difference in 11*β*-HSD 1 activities between stressed rats and controls. However, Matsuo and Tsuji [17] showed that exposure to light caused a significant decrease in the movement of rats and that albino rats were especially averse to this. Therefore it may be possible that if exercise indeed increases skeletal muscle 11*β*-HSD 1 activities [96], then lower muscle use caused by less movement of rats exposed to continuous light *may* diminish it. There are many problems with this hypothesis, however, as (a) the small cage size made it unlikely that the differences in movement were significantly lower in rats subject to a normal light/dark cycle, since the limited space would have restricted movement in any case, and (b) other experiments conducted have shown different effects of exercise on 11*β*-HSD 1 activities; for example, Coutinho et al. [98] showed that voluntary exercise did not cause any significant difference in skeletal muscle 11*β*-HSD 1 activities (as the exercise in this experiment was voluntary, it was not deemed stressful). This makes sense as exercise has been shown to improve insulin sensitivities [99], whereas active GCs induced by 11*β*-HSD 1 decrease insulin sensitivities. One possible reason for this, however, is the concurrent increase in interleukin-6 (IL-6) with exercise due to spillover from muscle [96]. IL-6 has been shown to improve skeletal muscle sensitivity to insulin [100]. Still, further investigations may be necessary to determine the exact cause of the decrease in skeletal muscle 11*β*-HSD 1 activities.

Figures 15 and 16 indicate that the 11*β*-HSD 1 activities in GFS rats were not significantly different from stressed rats in both QF and AM, respectively. One possible reason for this, at least in the case of AM, is that stress had already caused a significant reduction in skeletal muscle 11*β*-HSD 1 activities, and that may have limited the extent for any further reduction caused by GA. 

## 4. Conclusion and Future Work

*Conclusion*. This experiment indicated that when exposed to continuous light at an intensity of 300–400 lux (continuous, moderate-intensity stress), Sprague-Dawley (SD) rats began to adapt to the stressor at approximately 17 days, as indicated by decreases in systolic blood pressure (SBP). As such, at the end of the four-week treatment period, there appeared to be no significant changes in many of the analysed parameters in stressed rats compared to controls. These include serum glucocorticoid (GC) and catecholamine levels, blood glucose and serum insulin levels, insulin sensitivities and SBP. However, a significant increase in 11*β*-HSD 1 activities was still apparent in the kidney, while a significant increase in 11*β*-HSD 2 activities was apparent in the liver. This was thought to be some of the “left-over” effects of the stress response. The abdominal muscle on the other hand displayed a significant decrease in 11*β*-HSD 1 activities with stress, contrary to many other studies. Overall, continuous moderate-intensity stress caused by light exposure did not cause any significant long-lasting adverse metabolic effects with regard to the parameters under investigation.

Glycyrrhizic acid (GA) did not induce any significant changes in many of the parameters analysed in this experiment, including serum GC and catecholamine levels, blood glucose and serum insulin levels, and insulin sensitivities. The lack of a significant effect on glucose levels and insulin sensitivities was accounted to the fact that much of the beneficial effects of GA with regard to glucose metabolism (that have been shown in many of the previous experiments done in our laboratory) may have gone into negating the adverse effects of stress prior to adaptation taking place. If this is true, it also suggests that GA would bring these parameters back to control levels faster, since the deviations from normal physiological levels would be less with GA feeding. This indicates the ability of GA to enhance the rate of adaptation. Prior to adaptation, the opposing effects of catecholamines on GA-induced inhibition of 11*β*-HSD 1 in certain tissues (especially the liver) may have also played a part in the observed lack of a significant GA-induced effect on the aforementioned parameters. 

Effects of GA were, however, still apparent on the activities of 11*β*-HSD in some tissues. GA significantly reduced renal 11*β*-HSD 1 activities compared to stressed rats and subcutaneous adipose tissue (SAT) 11*β*-HSD 1 activities compared to control rats. It also significantly reduced hepatic 11*β*-HSD 2 activities compared to stressed rats and visceral adipose tissue (VAT) 11*β*-HSD 2 activities compared to control rats. Overall, the effects of GA on stress could not be fully elucidated as rats had already adapted to the stressor. However, the ability of GA to normalize the increase in 11*β*-HSD 1 activities caused by stress in the kidney may provide one possible mechanism by which it enhances the rate of adaptation, as many of the aberrations in glucose metabolism and subsequent metabolic abnormalities would occur as a result of increased 11*β*-HSD 1 activities involved in glucose production.

Finally, within the dosage and treatment time, GA did not significantly increase SBP beyond the changes already induced by stress at any point of the four-week treatment period.
